# Appraising Quality of GP Discharge Letters Completed by the Liaison Team for Patients Reviewed by South of Tees Liaison Team

**DOI:** 10.1192/bjo.2025.10664

**Published:** 2025-06-20

**Authors:** Heba Saeed, Ramanand Badanapuram, Sahana Sridhar, Sagrika Nag

**Affiliations:** Tees Esk and Wears Valley NHS Trust, Middlesbrough, United Kingdom

## Abstract

**Aims:** Discharge letters from liaison services to General Practitioners (GPs) are critical for ensuring continuity of care. These letters should contain essential clinical information that aids GPs in managing patients post-discharge. The purpose of this audit is to assess the quality and completeness of discharge letters from South of Tees liaison services and ensure they meet the necessary standards for effective communication.

**Methods:** Obtain a list of all patients discharged from South Liaison team during the period of 1 September to 30 September.

Randomized selection of 35 GP letters.

Establish availability of discharge letter on the electronic systems.

Appraise discharge letters for patients discharged using audit tool.

**Results:** Discharge letters on CITO: Only 40% of the letters were available on electronic system. RED.

Lack of GP credentials on the letter: 44% had a clear address and name of the GP practice. RED.

Date of referral to and date of discharge from PSL services: 18% of the letters had a clear mention regarding date of referral while none of the letters mention date of discharge. RED.

Reason for referral: 62% of the available letters had a clear reason for referral. AMBER.

Background history: 44% of the letters noted background history, with majority of them only mentioning psychiatric history. RED.

Summary of clinical assessment: 19% had a section for summary of clinical assessment which is useful to the user, especially the GP, to have an overview regarding psychiatry liaison service involvement. RED.

Risk assessment: 75% of the letters had an easy to identify risk assessment with majority not covering all 3 main groups of risks which include risk of self-harm/suicide, risk to others and risk from others. AMBER.

Diagnosis: 66% of the letters had a clearly defined diagnosis or clinical impression which were at times (43%) not concise. AMBER.

Medication: Only 12% of the letters had a list of medications and any changes to medication by psychiatry liaison services clearly documented. RED.

Actions for GP: Only 29% of the letters had an identifiable list of actions for the GP to undertake. RED.

**Conclusion:** The audit highlighted that lack of a local service specific guidance and a lack of a standardized GP format led to marked variability, lack of consistency and missing of vital information from GP discharge letters; furthermore it became apparent that some letters were not uploaded to the electronic system.

